# Equid Alphaherpesvirus 1 Modulates Actin Cytoskeleton and Inhibits Migration of Glioblastoma Multiforme Cell Line A172

**DOI:** 10.3390/pathogens11040400

**Published:** 2022-03-25

**Authors:** Michalina Bartak, Marcin Chodkowski, Anna Słońska, Marta Grodzik, Jarosław Szczepaniak, Marcin W. Bańbura, Joanna Cymerys

**Affiliations:** 1 Division of Microbiology, Department of Preclinical Sciences, Institute of Veterinary Medicine, Warsaw University of Life Sciences, 02-786 Warsaw, Poland; marcin_chodkowski@sggw.edu.pl (M.C.); anna_slonska@sggw.edu.pl (A.S.); marcin_banbura@sggw.edu.pl (M.W.B.); joanna_cymerys@sggw.edu.pl (J.C.); 2 Military Institute of Hygiene and Epidemiology, Kozielska 4, 01-163 Warsaw, Poland; 3 Department of Nanobiotechnology, Institute of Biology, Warsaw University of Life Sciences, 02-786 Warsaw, Poland; marta_grodzik@sggw.edu.pl (M.G.); jaroslaw_szczepaniak@sggw.edu.pl (J.S.)

**Keywords:** EHV-1, oncolytic virus, actin cytoskeleton, glioblastoma multiforme, cancer

## Abstract

Equid alphaherpesvirus 1 (EHV-1) causes respiratory diseases, abortion, and neurological disorders in horses. Recently, the oncolytic potential of this virus and its possible use in anticancer therapy has been reported, but its influence on cytoskeleton was not evaluated yet. In the following study, we have examined disruptions in actin cytoskeleton of glioblastoma multiforme in vitro model—A172 cell line, caused by EHV-1 infection. We used three EHV-1 strains: two non-neuropathogenic (Jan-E and Rac-H) and one neuropathogenic (EHV-1 26). Immunofluorescent labelling, confocal microscopy, real-time cell growth analysis and Oris^TM^ cell migration assay revealed disturbed migration of A172 cells infected with the EHV-1, probably due to rearrangement of actin cytoskeleton and the absence of cell projections. All tested strains caused disruption of the actin network and general depolymerization of microfilaments. The qPCR results confirmed the effective replication of EHV-1. Thus, we have demonstrated, for the first time, that EHV-1 infection leads to inhibition of proliferation and migration in A172 cells, which might be promising for new immunotherapy treatment.

## 1. Introduction

Cancer remains the most dangerous and widespread non-contagious disease that affects people around the world. Until now, traditional anti-cancer therapies such as chemotherapy, radiotherapy, resection, and radiosurgery have provided hope for the patient, but have often resulted in severe cytotoxicity. New solutions, such as anti-cancer therapy using viruses, is a promising method of treating many types of cancer. Since the introduction of the virus into the body stimulates an adaptive immune response, this strategy is considered as an immunotherapy [1]. It uses both genetically modified, and naturally occurring viruses that selectively replicate and lead to the degradation of cancer cells without affecting normal cells [2].

Equid alphaherpesvirus 1 (EHV-1) is a member of the subfamily *Alphaherpesvirinae* and a major factor of the upper respiratory tract diseases, mare abortion, and neuropathies in horses. Like other alpha-herpesviruses (e.g., human herpesvirus type 1(HHV-1), human herpesvirus type 2 (HHV-2), varicella-zoster virus (VZV), pseudorabies virus (PRV), bovine herpesvirus type 1 (BHV-1)), it exhibits neurotropism and causes latent infection of the host cells [3,4]. A virus that adversely affects animal cells has been shown to be potentially helpful for humans. Courchesne et al. (2012) discovered the oncolytic potential of this virus and set out to investigate its effect on glioblastoma multiforme cells. One of the results of this groundbreaking study was the understanding of how the virus enters the glioblastoma cell via the Major Histocompatibility Complex (MHC-1) receptor [5]. However, the changes in the cytoskeleton structure of A172 cells (human glioblastoma cell line) have not yet been examined. By expanding knowledge in this area, cytoskeleton can be used as a new immunotherapeutic target for oncolytic viruses.

The following structures are responsible for the movement of cancer cells: invadopodia, lamellipodia, and filopodia and depend on the reorganization of the actin cytoskeleton. On the other hand, actin-binding proteins (ABP) are responsible for controlling the polymerization and depolymerization process. Actin is a highly conserved protein that is found extensively in eukaryotic cells. There are two main forms—globular G-actin (monomer) and fibrillar F-actin (long-chain polymer) [6]. The state of dynamic equilibrium between F- and G-actin results from changes in ABP in neoplastic cells, which results in incorrect regulation [7].

The host cells cytoskeleton is also a crucial during replication and spreading of viruses [8]. One of the first components of the cytoskeleton that the virus encounters in the process of penetrating the cell is actin network [9]. Filamentous actin is responsible for some of the key functions of the cell such as cell integrity, formations of stress fibers, cell protrusions (filopodia and lamellipodia), the perinuclear and cell cortex. In context of viral replication, the most important feature, is polymerization of actin filaments which motors the crawling locomotion of eukaryotic cells and supports spread of viral particles. The layer of dense microfilaments beneath the plasma membrane (the cortical actin meshwork) is responsible for creating a physical barrier against viral infection. However, viruses use this structure to facilitate the processes of entry and post-entry pathways. In addition, actin filaments also influence the organization of surface receptors and membrane structures that mediate viral attachment, highlighting the complex and essential role of actin in early infection [9,10,11]. In case of herpesviruses, it is known that they interact with actin filaments at various stages of their replication cycle, causing polymerization or fragmentation of filaments [12,13,14,15,16]. EHV-1 influence on cytoskeleton was previously examined in Vero cells (african green monkey kidney), ED cell culture (equine dermis cells), and primary murine neurons. The results clearly showed that changes that occur under EHV-1 infection are depended on cells origination [17,18,19,20].

There are only a few data on alpha-herpesviruses infection and its effect on the cytoskeleton of glioblastoma multiforme cells. Most of the analysis focused on early-stage, in vitro studies investigating the aspect of genetic and molecular alterations in protein or marker expression. Clinical trials using herpesviruses, in particular HHV-1 (human herpesvirus type 1), have been conducted for years [21,22]. Application of EHV-1 as an oncolytic virus in the treatment of glioma cell lines was already proposed by Courchesne et al. (2012) [5] and White and Frampton (2013) [23]. In our previous study, we have demonstrated that animal virus EHV-1 productively replicated in the human adenocarcinoma cell line (A549) without the need for adaptation. Real-time PCR analysis and immunofluorescence assay showed that EHV-1 infected and caused lysis of human lung cancer cells [20].

In the present study, we have evaluated the oncolytic potential of three EHV-1 strains on glioblastoma multiforme cells. Our results determined that EHV-1 productive infection presumably inhibits the migration of A172 cells by annihilation of actin cytoskeleton. Inhibition of A172 cell migration by EHV-1 infection may be an essential discovery relevant to glioblastoma multiforme therapy limiting tumor metastasis.

## 2. Results

### 2.1. EHV-1 Modulates Actin Cytoskeleton in A172 Cells

**Figure 1:**
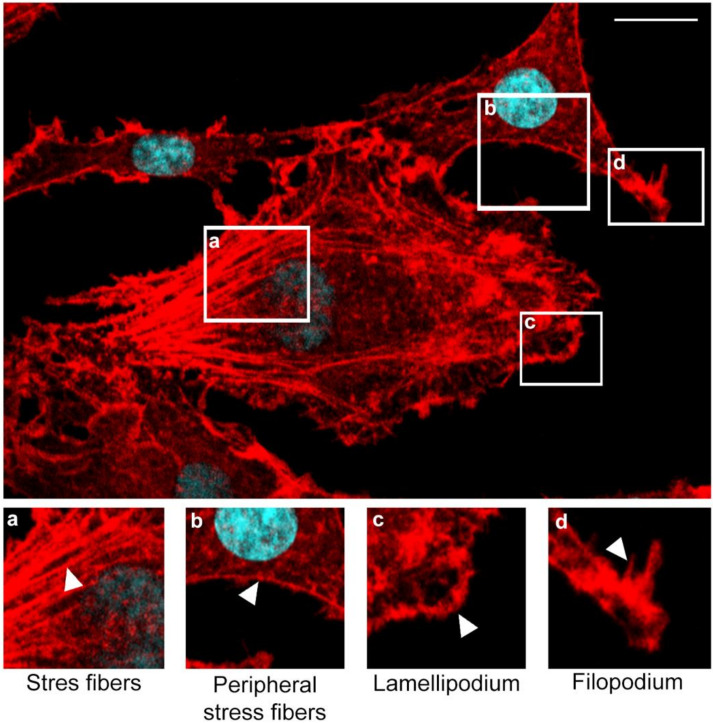
Merge image from confocal microscope presents actin cytoskeleton network morphology in mock-infected glioblastoma multiforme cells. Various forms of actin fibers structures were presented and highlighted by white arrowheads: stress fibers (**a**), peripheral stress fibers (**b**), lamellipodium (**c**), filopodium (**d**). Actin cytoskeleton—red fluorescence, nuclei—blue fluorescence. Objective magnification ×60. Scale bar 10 μm.

Immunofluorescence analysis of cellular distribution of actin filaments in Jan-E, Rac-H and EHV-1 26 -infected A172 cells were performed using confocal microscopy at 24, 48, 72, 96, and 168 h post infection (hpi). Mock-treated cells were mostly morphologically unequal, some multinucleated with numerous projections such as filopodia and lamellipodia of actin network (Figure 1c,d). Also, different forms of these fibers were able to be discerned, such as dorsal and abdominal stress fibers (Figure 1a) and peripheral stress fibers (Figure 1b).

**Figure 2:**
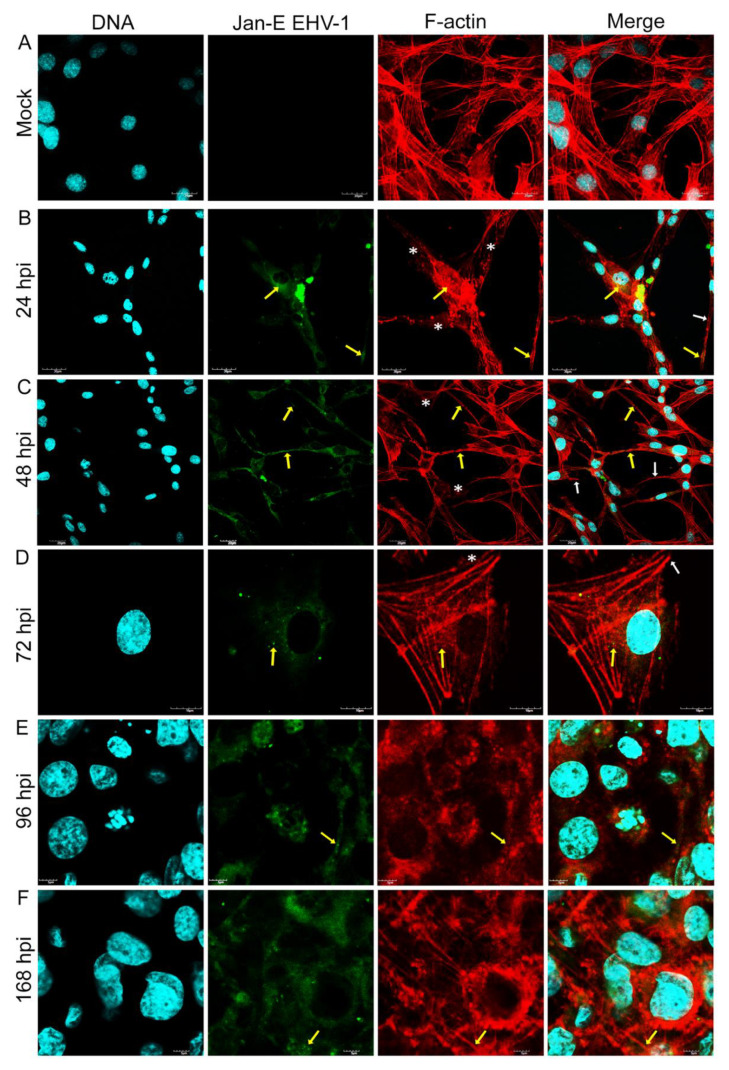
Actin cytoskeleton network morphology of uninfected glioblastoma multiforme cells (**A**) and during Jan-E EHV-1 infection (**B**–**F**). Representative confocal images of A172 cells obtained at 24, 48, 72, 96 and 168 hpi. Yellow arrows point the colocalization of viral antigens with actin network. White arrows indicate actin filament protrusions or structures, and white asterisks mark the growth of lamellipodia. Actin cytoskeleton—red fluorescence, viral antigens—green fluorescence, nuclei—blue fluorescence. Objective magnification ×60.

**Figure 3:**
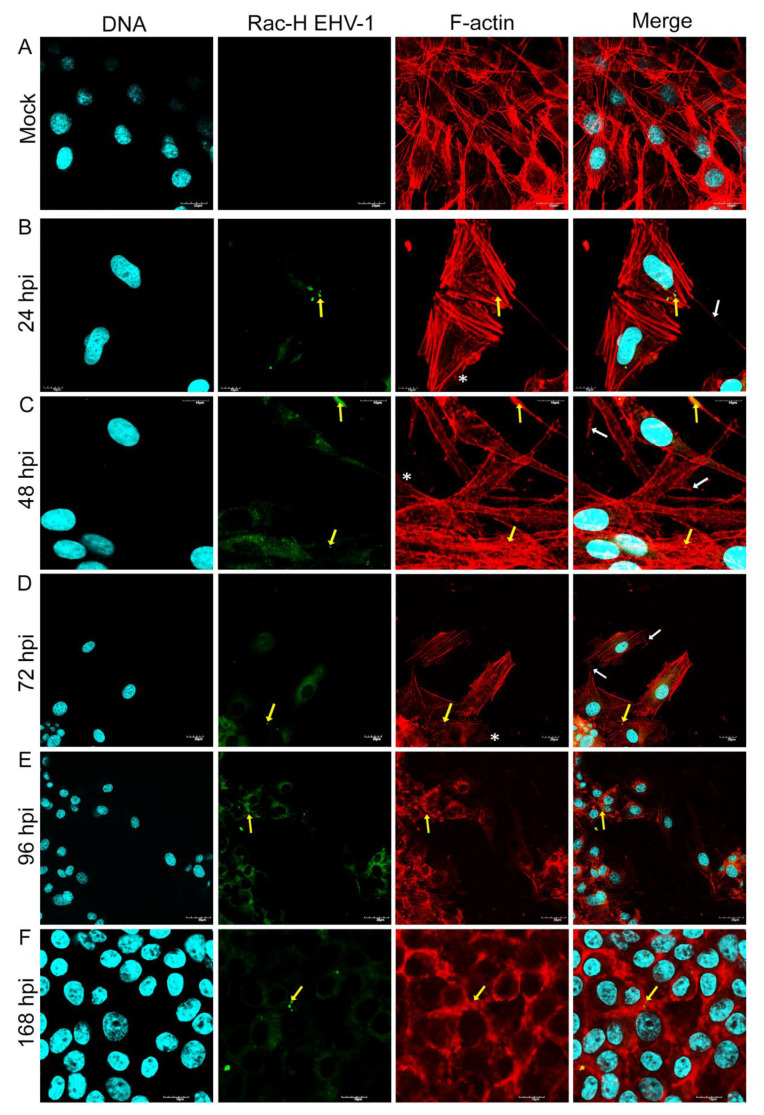
Actin cytoskeleton network morphology of uninfected glioblastoma multiforme cells (**A**) and during Rac-H EHV-1 infection (**B**–**F**). Representative confocal images of A172 cells obtained at 24, 48, 72, 96, and 168 hpi. Yellow arrows point the colocalization of viral antigens with actin network. White arrows indicate actin filament protrusions or structures, and white asterisks mark the growth of lamellipodia. Actin cytoskeleton—red fluorescence, viral antigens—green fluorescence, nuclei—blue fluorescence. Objective magnification ×60.

**Figure 4:**
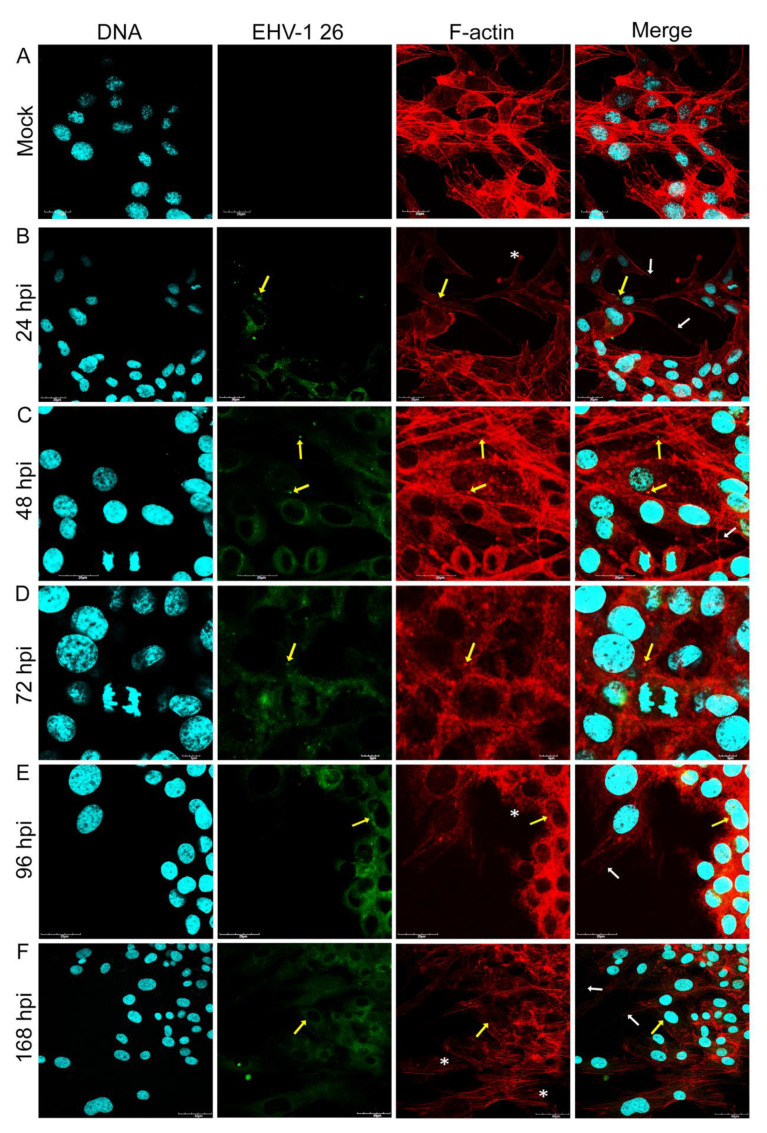
Actin cytoskeleton network morphology of uninfected glioblastoma multiforme cells (**A**) and during EHV-1 26 infection (**B**–**F**). Representative confocal images of A172 cells obtained at 24, 48, 72, 96 and 168 hpi. Yellow arrows point the colocalization of viral antigens with actin network. White arrows indicate actin filament protrusions or structures, and white asterisks mark the growth of lamellipodia. Actin cytoskeleton—red fluorescence, viral antigens—green fluorescence, nuclei—blue fluorescence. Objective magnification ×60.

**Figure 5:**
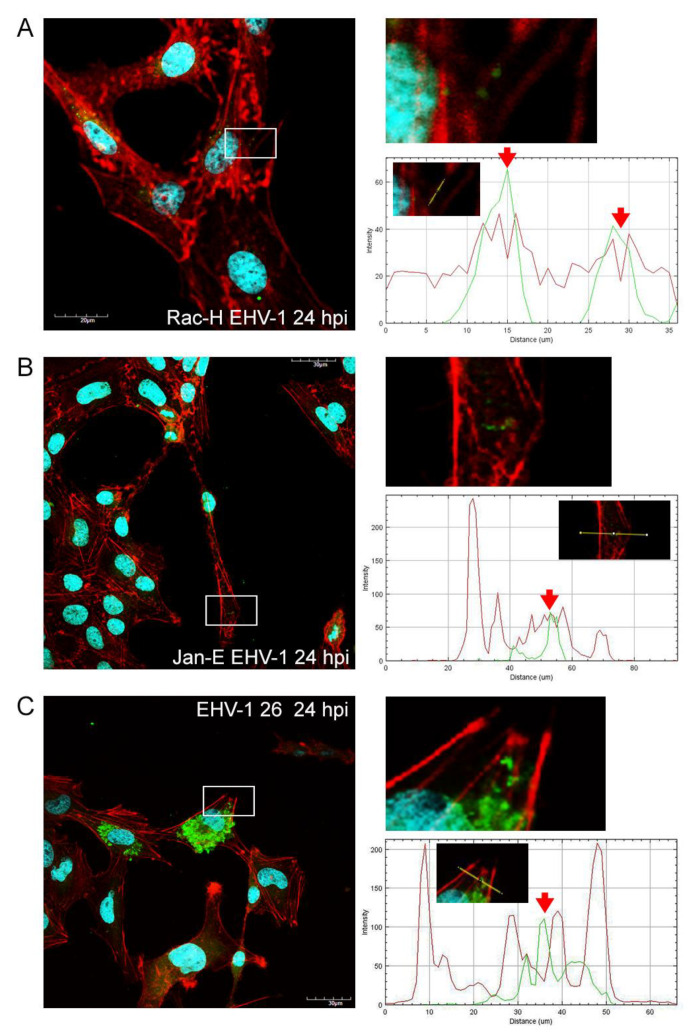
Representative confocal images of actin cytoskeleton network morphology in glioblastoma multiforme cells infected with EHV-1 Rac-H(**A**), Jan-E(**B**) and EHV-1 26(**C**) (24 hpi). White frames highlight the locations of viral antigen in actin filaments. Profile plot of fluorescence signal intensities along the yellow lines signify the colocalization of actin and viral antigen. The red arrow indicates the overlap of emission waves. Objective magnification ×60.

After infection of glioblastoma A172 cells with all three EHV-1 strains, the actin cytoskeleton was reorganized, fragmented, and depolarized. These changes were firstly manifested by the enhanced formation of long projections (Figure 2B, Figure 3B and Figure 4B—white arrows) in which the viral antigen was present at early hours post infection (24 hpi) (Figure 2B, Figure 3B and Figure 4B—yellow arrows). Additionally, the presence of viral antigen in actin filaments was confirmed by fluorescence colocalization analysis performed with the ImageJ program (Figure 5). However, increasing number of cells’ projections used during virions transport and intercellular bridges were visibly enhanced in case of the infection with Jan-E strain (Figure 2B,C white arrows). Especially at 48 hpi, many fine filopodia bridges (nanotubular bridges) have been detected (Figure 2B white arrows).

Following infection with Rac-H, the formation of long thin filopodia and actin intercellular bridges (Figure 3B,C white arrows) has been distinguished. Also, lamellipodia-like structures have been present at leading edges of the cells. (Figure 3B,C white asterixis). Intriguingly, EHV-1 26 contributed to the formation of lamellipodia (Figure 4B white asterixis) and long thin intercellular bridge (Figure 4B white arrow) in A172 cells in the initial hours after infection. After 72 hpi, such numerous protrusions/structures of actin filaments, in case of Rac-H and Jan-E infection, were not observed. Stress fibers were well defined and more polarized in cortical area (Figure 2D and Figure 3D). More lamellipodia were visible (Figure 2D and Figure 3D white asterixis) than thin, short filopodia formation on leading edges (Figure 2D and Figure 3D white arrows). In contrast, cells infected with EHV-1 26 strain regain the ability to form actin rich structures at 96 and 168 hpi (Figure 4E,F white arrows and asterixis). Moreover, the actin repolarized and defined the stress fibers in the A172 cells (Figure 4F).

Another important observation was the gradual depolymerization of actin filaments in the peripheral region, the condensation of actin filaments around the perinuclear space, and the disappearance of F-actin at the sites of cell cortical at 48 hpi and 72 hpi for EHV-1 26 (Figure 4C,D). These changes in cells were also seen for Jan-E at 72 hpi and alike for Rac-H strain (Figure 2E,F and Figure 3E,F). Moreover, there was an accumulation of viral antigen at the site of F-actin condensation around the nucleus. This phenomenon increased after 96 hpi and was mainly manifested in cells infected with Jan-E and Rac-H strains (Figure 2E,F and Figure 3E,F).

### 2.2. Cytopathic Effect Manifest in A172 as Plaques

**Figure 6:**
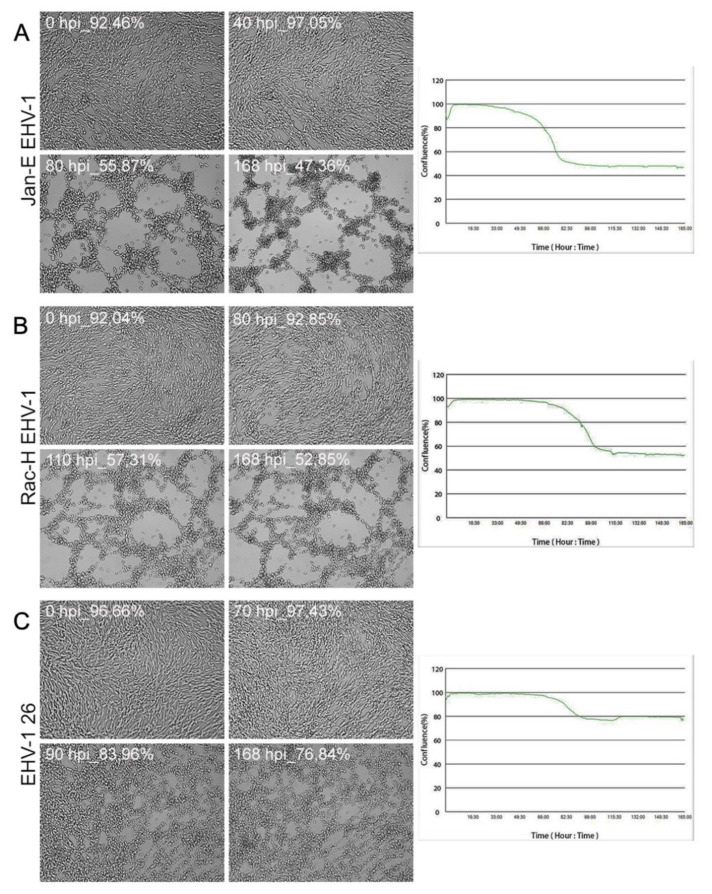
Morphological changes in A172 cells infected with EHV-1 strains (**A**–**C**). Cells were observed for 168 h through live imaging analyzer JuLI™ Br. CPE manifestation occurred as plaque formation and focal degeneration, confirmed by growth curve. Objective magnification ×40.

During real-time cell growth analysis (JuLI™ Br) performed on all EHV-1 strains, the disturbances in the confluence rate have been observed and the presence of cytopathic effect of infection. The given results are snapshots from specific time intervals representing post-infection changes. At first stage of infection with all EHV-1 strains, the confluence [%] was estimated above 92%. The cytopathic effect, which results from productive replication, was manifested by plaque formation. In the case of EHV-1 26 infection, the plaques formed at 90 hpi, and the confluence rate dropped to 83% (Figure 6C). For EHV-1 Jan-E, the cytopathic effect was visible at 80 hpi (Figure 6A,B). The confluence rate was drastically lower—47%, contrary to EHV-1 26.

### 2.3. EHV-1 Inhibits Cell Migration

**Figure 7:**
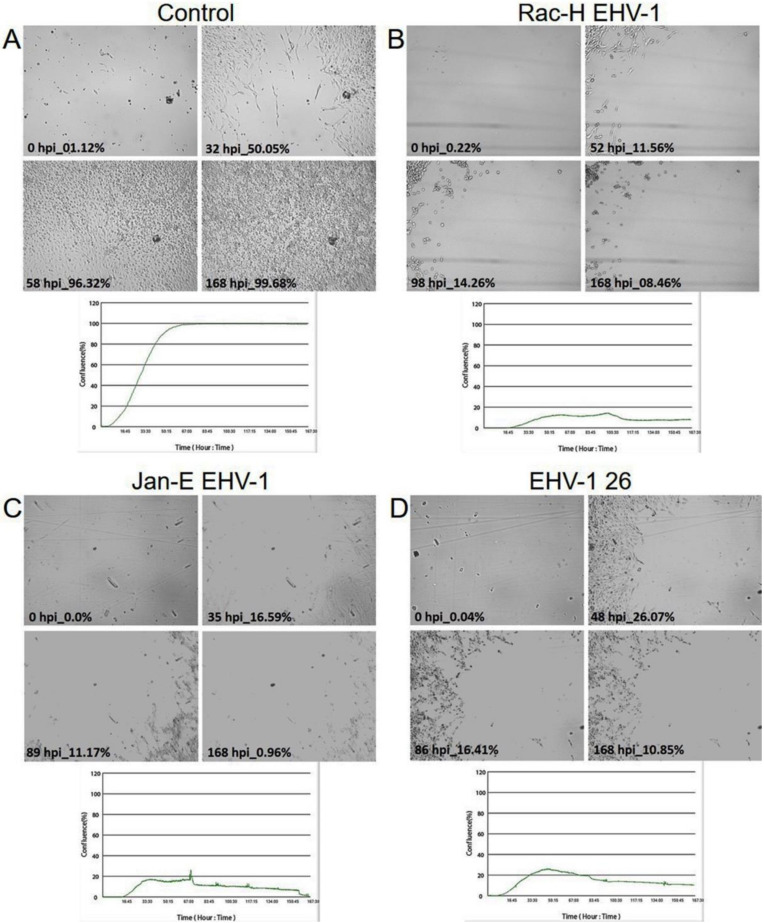
Cell migration assay of A172 cells infected with EHV-1 strains (**B**–**D**). Cells were observed for 168 h through live imaging analyzer JuLI™ Br. The camera was focused on the field as shown in the diagram in Figure 8 (materials and methods section). The confluence plots show the percentage of overgrowth of the cell-free area relative to the control (**A**). Objective magnification ×40.

Inhibition of cell migration was noted for all EHV-1 strains. The morphology characteristics and time required for cell movement disruption were distinguished depending on the strain. For the control, uninfected A172 cells, the confluence was already 50% at 32 h and almost 100% at 58 h (Figure 7A).

However, in cells infected with EHV-1 Rac-H and EHV-1 Jan-E (Figure 7B,C), migration was inhibited as early as 52 hpi and 35 hpi, respectively. Also, confluence did not reach 20% in both cases. Cells infected with EHV-1 Rac-H were rounded and did not form cell protrusions. At 100 hpi, cell’s death was observed, and the growth curve showed a decrease in confluence to 8%. Similarly, in the case of infection with the EHV-1 Jan-E strain, the cells had stopped dividing after 67 hpi, which was manifested as a decrease in confluence—below 15%. After 168 hpi, the cell culture confluence had gradually reached less than 1%. The morphology of the cells during the EHV-1 Jan-E strain infection was different from those infected with EHV-1 Rac-H. Up to 89 hpi, cells had been forming multiple protrusions and intercell bridges. After that time, change in the cytoplasm ratio to the nucleus was observed, and a rounding of the cells was visible.

In contrast, EHV-1 strain 26 (Figure 7D), a neuropathogenic strain, clearly maintained cell growth for 168 hpi. The cells morphology was characterized by the formation of distinct, long filopodia. The confluence ratio was also maintained at a high level. Cells proliferated up to 48 hpi, reaching a confluence of 26%, and then a gradual decrease in confluence was detected. At 168 hpi, the confluence took the highest value among the EHV-1 strains, reaching almost 11%.

### 2.4. EHV-1 Replicates in Glioblastoma Cells

Real-Time PCR analysis confirmed the effective replication of all EHV-1 strains in A172 cells. The obtained results were statistically significant *(p <* 0.05–*p* < 0.001). All used strains achieved the most efficient replication at 72 and 96 hpi (Figure 9A–C). The EHV-1 Jan-E maintained high and similar levels of replication throughout the process. Moreover, contrary to the other strains, Jan-E replication was the highest at 24 and 48 hpi (Figure 9B). It was also noted that viral DNA concentration remained at high level, both in the cell medium and in cells (Figure 9B). The reference strain, EHV-1 Rac-H, obtained the highest replication level at 72 and 96 hpi. (Figure 9A). However, Rac-H exhibited similar replication kinetics as EHV-1 26. (Figure 9A,C). Within the 168 h post infection, the DNA level of all EHV-1 strains remained relatively high (Figure 9A–C).

## 3. Discussion

The changes occurring in the cell cytoskeleton are closely related to the cell’s physiological state and the progression of tumor development [24]. The three main components of the cytoskeleton are microtubules (MT), microfilaments (actin filaments, MF), and intermediate filaments (IF). This structure undergoes continuous remodeling and is responsible for migration, intracellular transport, and movement of organelles between cells [25]. In the context of our research, we focused on EHV-1—microfilaments interaction. Actin filaments are involved in the internalization of membrane vesicles, protrusion of filopodia and lamellipodia, chemotaxis, cytokinesis and, most importantly, cell migration [10]. They are also utilized by cells undergoing carcinogenesis and play an essential role in their migration. Tumor metastasis is still a vast problem, especially in malignant brain cancers. This phenomenon is remarkably relevant to GBM and is associated with increased actin polymerization so that the cells form protrusions and can migrate beyond the tumor mass area [26]. Spatially controlled assembly of actin filaments generates lamellipodia, filopodia, and pseudopodia, which are used by cells to explore the extracellular space during invasion and metastasis [27]. Taking it into consideration, we have discovered, for the first time, that EHV-1 inhibits migration of human glioblastoma cells (A172) by depolymerization of the actin cytoskeleton. This finding is crucial because GBM cells are notorious for their invasiveness, ability to develop resistance to chemo- and radiotherapy, and form secondary site tumors [26]. Previous studies conducted by Courchesne and Frampton (2011; 2013) confirmed that EHV-1 has many beneficial properties, such as a well-defined growth cycle that results in the death of infected cells and a lack of pre-existing immunity to the virus, that make it a potential new anti-cancer agent. They assessed the oncolytic properties of the designed EHV-1 strains against malignant glioma—recombinant EHV-1 LacZ derived from RacL11 (L11ΔgIΔgE) with use of MHC-I as a cell entry receptor [5,23].

Data on alpha-herpesvirus infection and its effect on the cytoskeleton of glioblastoma multiforme cells are scarce. Most analyses focus on the preliminary research phase, in vitro studies in which genetic and molecular changes regarding protein or marker expression are investigated. Herpesvirus, adenovirus, vaccinia virus, reovirus, parvovirus, poliovirus, measles virus, replicating retrovirus vector, and Newcastle disease virus (NDV) are presently being tested in malignant glioma patients for safety and efficacy at different clinical phases. For years, clinical trials have been conducted using herpesviruses, particularly HSV-1 derivatives—G207, G47Δ, rQNestin34.5v.2, or C134. The G47Δ is the first OV approved for CNS tumor therapy and is a registered treatment for malignant glioma in Japan’s MHLW [21,22]. Unfortunately, no studies have described the changes occurring in the actin cytoskeleton of EHV-1-induced glioblastoma multiforme cells to date.

Our study provided the evaluation of the equid alphaherpesvirus 1 infection by confocal microscopy, JuLI™ Br live-time imaging, and Oris™FLEX kit, which revealed several changes in glioblastoma cells morphology and movement in case of all EHV-1 strains used. Analyzing the obtained data, we have observed not only the reorganization and depolarization of the actin cytoskeleton after infection with EHV-1 strains, but we have also noted the inhibition of cell motility. The changes observed by confocal microscopy were manifested by the formation of elongated actin protrusions—filopodia, intercellular bridges, and even cytoneme-like structures up to 48 hpi. It can be inferred that EHV-1 uses actin filaments as channels to transport virions between adjacent cells without driving the cell to the lysis pathway. Interestingly, during infection with Rac-H and Jan-E, cells have lost the ability to form protrusions after 48 hpi with visible depolymerization of actin and disruption of the filamentary network. In contrast, cells infected with EHV-1 26 after 72 hpi were characterized by the emergence of new protrusions with present viral antigen.

Particularly in the context of GBM, tunneling nanotubes (TNTs) are significant actin structures. These structures, which can be easily mistaken for filopodia, are responsible for cell-to-cell communication, transmission of signaling molecules in dendritic cells, small molecules (ions), macromolecules (proteins, nucleic acids), or even cell organelles (mitochondria, lysosomes) [28,29]. These structures are present in small amounts in cells of healthy, adult individuals. With the onset of disease and inflammation, their amount increases significantly. TNTs proliferate in response to increased oxidative stress caused by viral, bacterial infection, prions, or the process of carcinogenesis. Then, bacterial/viral protein, membrane fragments, or organelles may be exchanged [30].

Considering that TNTs are activated in pathological states, it could be speculated that their increased amount appears in the initial times after EHV-1 infection and is controlled by the virus to facilitate the spread of progeny virions between cells. Subsequently, when the processes of polarization and depolarization of actin particles are completely disrupted by infection, GBM cells are unable to form protrusions which prevent them from migrating and exchanging signaling particles or metabolites.

Thus, it could be assumed that appropriate modulation of TNT structures and other mentioned actin structures by an agent such as EHV-1 infection could somehow reduce tumor growth and metastasis. This mechanism could be combined with the idea described in the new research investigating adaptation of TNTs by GBM cells to spread cell resistance to temozolomide (TMZ) and radiotherapy (IR) treatments. GMB utilizes structures as “highways” through which TMZ resistance can be spread via TNT-mediated transfer of MGMT (DNA repair enzyme O^6^-methylguanine-DNA methyltransferase) [30]. Thus, TNTs become a therapeutic target, and EHV-1 infection allows limiting/stopping the proliferation of these structures.

Obtained results were confirmed by Real-Time PCR replication analysis. The EHV-1 26 DNA copy number was significantly higher in samples collected from cells after 48 hpi. It could be assumed that EHV-1 26 in human GBM cells undergoes a specific latency state typical for herpesviruses. A specific knock-down of latency-related gene transcripts (LATs) should be considered in the context of potential use and further research of this strain. A different phenomenon was observed for Rac-H and Jan-E strains. In this case, after 48 hpi, the GBM cells do not regain the ability to form protrusions or intercellular bridges. The disappearance of actin filaments in the area of focal adhesion sites may indicate impaired migratory ability. 

This hypothesis was confirmed by the analysis performed using the Oris™FLEX cell migration assessment kit. Analyzing the wound healing percentage diagrams and snapshots of real-time observation (JuLI™Br) of cell culture revealed that infection with all EHV-1 strains decreased the migration activity by inducing malformation in the actin cytoskeleton of the A172 cells. It is likely that EHV-1-infected glioma cells, particularly Jan-E and Rac-H strains, depolymerize actin filaments and thus cannot form cell protrusions that allow cells to move. The changes in actin cytoskeleton morphology compared to other neural cells of normal phenotype maybe since the hypoxic (hypoxia) environment coordinates changes in cytoskeleton dynamics, thus promoting migration of these cells. In addition, cell migration is often associated with excessive dynamic polymerization of actin at the leading edge of cells at focal adhesion sites, especially in cell protrusions [26]. Those EHV-1 strains modify actin polymerization, initially promoting migration and then gradually affecting depolymerization of the actin cytoskeleton leading to lysis of tumor cells, thus blocking proliferation of A172 human glioblastoma multiforme cells. It was seen in the gradual lowering of confluence percentage on the wound healing diagram. It is crucial for GBM cells to rapidly polymerize actin, especially at the leading edges of the cell, to form protrusions. The other therapeutic strategies were examining the hypoxic environment and overexpression of the CTTN gene to disable this mechanism [26]. 

The latest studies performed by Chodkowski et al. (2021) also revealed the possible oncolytic potential of EHV-1 Jan-E. The results indicated productive replication in the human adenocarcinoma cell line (A549) without additional adaptation of EHV-1. Real-time PCR analysis and immunofluorescence assay showed that EHV-1 infected and caused lysis of human lung cancer cells [20]. Compering confocal analysis with our findings, we also have noted that in cells that did not undergo lysis accumulation, of viral antigens in the cytoplasm, accompanied by increased density of actin fibers, occurred. Despite different cell lines, comparable viral influence on actin cytoskeleton was detected. Also, a similar cytopathic effect was manifested in the A549 cell line, such as cell destruction, focal degeneration, and plaque formation. In the case of the A172 cell line, there were no sights of syncytia formation.

For the first time, the oncolytic potential of EHV-1 was described by Courchesne (2012), White and Frampton (2013), however, no observation of cytoskeletal or motile disturbances was investigated [5,23]. Detailed analysis of infection with a modified strain of EHV-1 (strain L11ΔgIΔgE, deletion of gI and gE glycoproteins, and embedded LacZ cassette) on five glioblastoma multiforme cell lines (A172, Hs 683, LN-18, SNB19, U251) was conducted to assess the oncolytic potential of the virus. EHV-1 productively infected four cell lines, and replication levels were positively correlated with glioma cell death. These findings were previously confirmed by Kurtz et al. (2010), who demonstrated that EHV-1 uses the MHC-1 complex to enter glioblastoma multiforme cells [31]. Moreover, in cell lines A172 and LN-18 high level of expression of this complex was detected, thus confirming the high sensitivity of these lines to EHV-1 infection (supported by MTT cell viability assay) [5,23].

In other studies, utilization the histone deacetylase (HDAC) inhibitor and valproic acid (VPA) enhanced EHV-1 L11ΔgIΔgE infection and caused limited glioma progression, thus improving the therapeutic potential of this virus. The study revealed that treating two human glioma cell lines (U251 and SNB19) with VPA resulted in significantly more efficient virus entry, establishing a practical approach in using OVs in synergistic combination therapies to maximize therapeutic outcome [23].

In conclusion, oncolytic virus (OV) therapy designates a promising new immune treatment strategy targeting cancer, particularly those located within the CNS. Oncolytic viruses can replicate in tumor cells but not in non-cancerogenic cells, resulting in lysis of the tumor-forming cell mass. Therefore, an effective viral vector used for potential therapeutic therapy should be safe, replicate rapidly, and lead to cell lysis. Many established researchers have conducted *in vitro* and *in vivo* studies using oncolytic viruses, including EHV-1, as vectors for anti-cancer therapy, combining viruses with drug therapy or using them alone [5,23,31,32,33]. In contrast to already used HHV-1, EHV-1 is an animal virus that might naturally avoid the problem of rapid virus elimination by antibodies or memory cytotoxic T lymphocytes resulting from pre-existing immunity [23,34].

Our studies revealed another favorable property of EHV-1 virus proving its oncolytic potential. All strains of EHV-1 have affected polymerization of the actin cytoskeleton, thus blocking proliferation and migration of A172 human glioblastoma multiforme cells. 

## 4. Materials and Methods

### 4.1. Cell Culture and Virus Maintenance

Human glioblastoma cell line A172 [ATCC CRL-1620™] was cultured in Dulbecco’s Modified Eagle Medium (DMEM) with 10% inactivated fetal bovine serum (FBS) and 40 mg/mL of streptomycin, penicillin (Gibco, Life Technologies, Waltham MA, USA) at 37 °C with 5% CO_2_. Three different EHV-1 stains were used: non-neuropathogenic, reference strain Rac-H (149th passage in ED cells); non-neuropathogenic Jan-E (12th passage in ED cells) strain isolated from aborted fetus (neuropathogenicity confirmed by PCR-RFLP neuropathogenic/non-neuropathogenic discrimination test) [35]; neuropathogenic EHV-1 strain (EHV-1 26, 15th passage in Vero cells) isolated from aborted fetus in Hungary in 2004 (neuropathogenicity confirmed by PriProET technique) [36]. All strains were obtained from the virus collection of the Virology Laboratory at Warsaw University of Life Sciences (SGGW). The viruses were propagated in equine dermal (ED) and Vero cell cultures grown in Eagle’s minimum essential medium (MEM) (Gibco Life Technologies).

### 4.2. Infecting Cells with EHV-1

A172 cells were infected with Rac-H, Jan-E, and EHV-1 26 strains (MOI = 1.0) for 60 min at 37 °C. After incubation, the inoculum was removed, washed with PBS and fresh culture medium was added. Subsequently, infected cells were incubated for 24, 48, 72, 96, and 168 h at 37 °C with 5% CO_2_.

### 4.3. Immunofluorescence Staining and Imaging

The direct immunofluorescence method was used to visualize cell structures and viral antigen. After incubation with the viruses, A172 cells were washed twice in PBS (Sigma-Aldrich, Darmstadt, Germany), then fixed in 3.7% PFA (paraformaldehyde) for 30 min. After fixation the cells were washed twice with PBS solution and further incubated with 0.5% Tween/PBS solution for 5 min at room temperature. Subsequently, the culture was washed twice with PBS solution. Actin filaments were stained with 50 µL of TRITC-labelled phalloidin conjugate (500 ng/mL; Sigma-Aldrich) and incubated for 60 min in a wet chamber. In order to visualize the viral antigen of EHV-1 strains FITC-labelled anti-EHV-1 antibody (1:16; RPK Gamakon conjugate; Mevak, Nitra, Slovakia) was used. Cells infected with EHV-1 strains were covered with the conjugate and incubated for 30 min at 37 °C. Cell nuclei were stained with Hoechst 33258 (Thermo Fisher, Waltham MA, USA) for 2 min. Afterward, cover slips were mounted on microscope slides using ProLong Gold Antifade Mounting Medium (Thermo Fisher). Confocal images were acquired in a confocal microscope (Fluoview FV10i, Olympus, Warsaw, Poland) and analyzed using FV10i software (Olympus), ImageJ (NIH Image, version 1.53a, Bethesda, MD, USA), and Adobe Photoshop CS6 software (Adobe Systems Incorporated, ver. 13.0, San Jose, CA, USA).

### 4.4. Quantitative Real-Time PCR

Viral DNA was isolated from samples collected after 24, 48, 72, 96, 168 hpi, separately from A172 cells and cell culture medium. The quantity of the EHV-1 DNA in all samples was determined using quantitative real-time PCR (qPCR) with fluorescent TaqMan probes, complementary for the sequence within the amplified products. For amplification of viral DNA, the primers specific for the glycoprotein B (gB) gene were used [gB-1, 5′–AAA CAA AGA GCG GAC CCT AT–3′; gB-2, 5′–TCC GTG AAA ATC TCG TTC TC–3′]. Amplification was performed with LightCycler TaqMan Master Kit (Roche Diagnostics, Mannheim, Germany). To obtain a standard curve, Jan-E EHV-1 strain was serially diluted in sterile deionized water to give ranges of CCID_50_ = 10^6^. DNA of the Rac-H strain was used as a positive control. The negative control was water included in the LightCycler TaqMan Master kit. Fluorescence level was measured at 530 nm wavelength. Tests were run on the LightCycler 2.0 instrument (Roche Diagnostics, Mannheim, Germany) according to the in-house quantitative method [3,37].

### 4.5. Real-Time Cell Growth Analise JuLi™ Br

To determine cellular growth and morphology of glioblastoma cells infected with various EHV-1 strains, JuLI ^TM^ Br Live Cell—system for bright-field analysis (NanoEnTek, Seoul, Korea 2015) was used. The A172 cell cultures that reached > 95% confluency were infected with Jan-E, Rac-H, and EHV-1 26 strains as previously described. Images were captured for 168 h with 30 min intervals. The results were obtained and analyzed using JuLI ^TM^ Br PC software. Uninfected cells were used as a negative control. All images were captured at a ×40 magnification.

### 4.6. Migration Assay—The Oris^TM^ Cell Migration Assembly Kit

The technology utilizes a 96-well plate populated with silicone-based cell seeding stoppers which exclude cells from attaching to a central zone.

**Figure 8:**
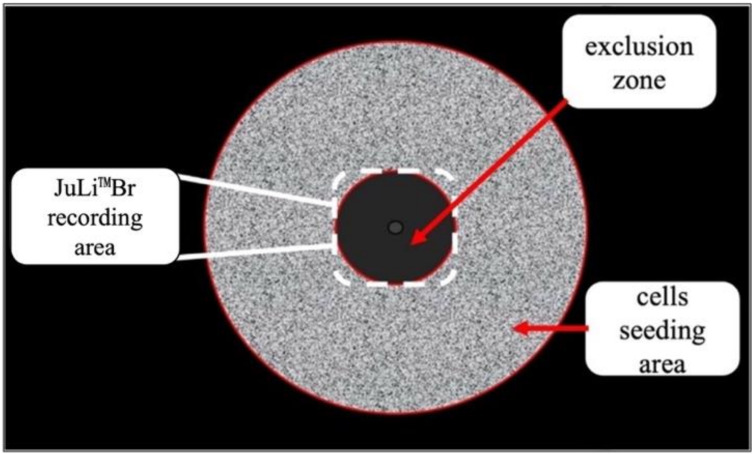
Area of JuLI™ Br camera recording and topography of the ORIS™ plate well.

**Figure 9:**
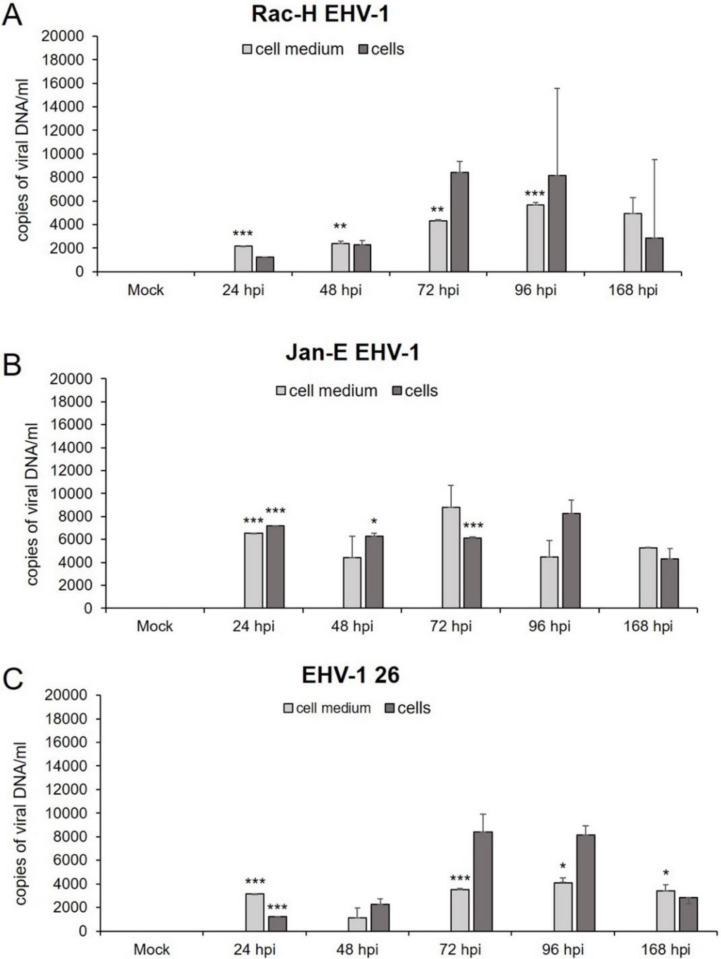
Viral DNA levels in glioblastoma multiforme A172 cell line infected with EHV-1 strains—Rac-H (**A**), Jan-E (**B**), and EHV-1 26 (**C**) by qPCR. The data were obtained from three independent biological experiments. Statistical comparisons were made between mock-infected and EHV-1-infected cells (*** *p* ≤ 0.001; ** *p* ≤ 0.01; * *p* ≤ 0.05).

A172 cells were seeded at a density of 5 × 10^4^ into an Oris™ 96-well plate with attached Oris™ silicon stoppers (Platypus Technologies, LLC, Madison, WI, USA). After the cells were seeded and allowed to adhere, the stoppers were removed to reveal a 2 mm diameter exclusion zone into which cells may then migrate [38]. After 18 h of attachment and reaching full confluency, the stoppers were removed. Then the cell cultures were infected with three EHV-1 strains for 1 h at 37 °C. Afterwards, the cell cultures were washed twice with PBS and cultured with fresh serum-free medium for 168 h at 37 °C, 5% CO_2_. The Oris™ plate was placed under the JuLI™ Br—Live Cell Movie Analyzer (NanoEnTek) (Figure 8). During the incubation period of the migration test, the JuLI™ Br camera has taken photos in 5 min intervals. The complete cell growth movie was cut to obtain from 0 to168 h post-infection with each EHV-1 strain to collect the final image. To evaluate the stage of migration inhibition, the wound healing diagrams were analyzed.

### 4.7. Statistical Analysis

The results were statistically evaluated by one-way analysis of variation (ANOVA) using the Student-Newman-Keuls multiple comparisons test and the Turkey-Kramer multiple comparisons test. These analyses were performed using GraphPad InStat^TM^ version 3.0 software (GraphPad Software Inc., San Diego, CA, USA). Statistical differences were interpreted as significant at *p ≤* 0.05, highly significant at *p ≤* 0.01, extremely significant at *p ≤* 0.001 and not significant at *p* > 0.05.

## Data Availability

The data presented in this study are available in the main text, figures, and tables.
